# The sonic hedgehog signaling pathway stimulates anaplastic thyroid cancer cell motility and invasiveness by activating Akt and c-Met

**DOI:** 10.18632/oncotarget.7228

**Published:** 2016-02-07

**Authors:** Ashley J. Williamson, Michelle E. Doscas, Jin Ye, Katherine B. Heiden, Mingzhao Xing, Yi Li, Richard A. Prinz, Xiulong Xu

**Affiliations:** ^1^ Rush Medical College, Rush University Medical Center, Chicago, IL, USA; ^2^ Department of Anatomy and Cell Biology, Rush University Medical Center, Chicago, IL, USA; ^3^ Department of General Surgery, Rush University Medical Center, Chicago, IL, USA; ^4^ Division of Endocrinology and Metabolism, Department of Medicine, The Johns Hopkins University School of Medicine, Baltimore, MD, USA; ^5^ Lester and Sue Smith Breast Center and Department of Molecular and Cell Biology, Baylor College of Medicine, Houston, TX, USA; ^6^ Department of Surgery, NorthShore University Health System, Evanston, IL, USA; ^7^ Center for Comparative Medicine, College of Veterinary Medicine, Yangzhou University, Yangzhou, Jiangsu Province, China

**Keywords:** sonic hedgehog signaling pathway, PI-3 kinase, c-Met, Snail, cell motility and invasion

## Abstract

The sonic hedgehog (Shh) pathway is highly activated in thyroid neoplasms and promotes thyroid cancer stem-like cell phenotype, but whether the Shh pathway regulates thyroid tumor cell motility and invasiveness remains unknown. Here, we report that the motility and invasiveness of two anaplastic thyroid tumor cell lines, KAT-18 and SW1736, were inhibited by two inhibitors of the Shh pathway (cyclopamine and GANT61). Consistently, the cell motility and invasiveness was decreased by Shh and Gli1 knockdown, and was increased by Gli1 overexpression in KAT-18 cells. Mechanistic studies revealed that Akt and c-Met phosphorylation was decreased by a Gli1 inhibitor and by Shh and Gli1 knockdown, but was increased by Gli1 overexpression. LY294002, a PI-3 kinase inhibitor, and a c-Met inhibitor inhibited the motility and invasiveness of Gli1-transfected KAT-18 cells more effectively than the vector-transfected cells. Knockdown of Snail, a transcription factor regulated by the Shh pathway, led to decreased cell motility and invasiveness in KAT-18 and SW1736 cells. However, key epithelial-to-mesenchymal transition (EMT) markers including E-cadherin and vimentin as well as Slug were not affected by cyclopamine and GANT61 in either SW1736 or WRO82, a well differentiated follicular thyroid carcinoma cell line. Our data suggest that the Shh pathway-stimulated thyroid tumor cell motility and invasiveness is largely mediated by AKT and c-Met activation with little involvement of EMT.

## INTRODUCTION

In mammals, the hedgehog pathway is regulated by three ligands: Sonic hedgehog (Shh), Indian hedgehog, and Desert hedgehog. In the absence of these ligands, the Shh pathway is inactive since the transmembrane receptor, Patched (Ptch) functions as a tumor suppressor to prevent Smoothened (Smo), a G-protein coupled receptor [1-3] from activating a family of oncogenic Gli transcription factors. Hedgehog binding to Patched leads to the uncoupling of Patched from Smo, subsequently leading to the activation of a signal cascade and the translocation of Gli into the nucleus to induce or repress gene expression [1-3].

The sonic hedgehog signaling pathway has been implicated in stimulating tumor cell motility and invasion in several types of malignancies [3]. One of the Gli1-regulated genes, SNAIL, can function as a transcription repressor for E-cadherin [4]. Loss of E-cadherin is a hallmark of the epithelial to mesenchymal transition (EMT). Intriguingly, Gli1 can directly bind the E-cadherin promoter and induce its expression [5, 6]. Whether the Shh pathway stimulates tumor cell invasiveness through EMT remains controversial. In addition, the Shh pathway may promote tumor cell invasiveness by cross-activating the PI-3 kinase pathway or induces metalloproteinase expression [7-15]. Disregulated Shh signaling correlates with the severity of the associated tumor and contributes to maintain metastatic behavior. Understanding the cross-talk between the Shh and other pathways will facilitate the discovery of novel therapeutics and the treatment of metastatic disease.

Improved understanding of genetic alterations such as identification of high frequency BRAF gene mutations in thyroid cancer offers new hope for promising target therapy. Undifferentiated anaplastic thyroid carcinoma (ATC) is almost always fatal, with a mean survival of 6 months [16]. However, significant challenges remain as drug resistance arises due to the transactivation of other signaling pathways or new gene mutations in the same pathway. Recent studies have identified thyroid cancer stem cells (CSCs) as a unique population (about 1-3%) that is highly tumorigenic and metastatic. Poorly differentiated or undifferentiated thyroid cancers contain a higher percentage of ALDH (aldehyde dehyrogenase)-positive CSCs than benign adenomas and well differentiated thyroid cancers. CSC-enriched ATC is highly invasive and metastatic due to hyperactivation of the PI-3 kinase pathway and c-Met [17, 18]. We and others have demonstrated that the Shh pathway is widely activated in thyroid neoplasm and promotes thyroid tumor cell proliferation [19-22]. Our further studies showed that the Shh pathway promotes the cancer stem-like cell phenotype of two anaplastic thyroid carcinoma cell lines, KAT-18 and SW1736 [23]. Here we report that the Shh pathway plays an important role in the tumor cell motility and invasiveness of anaplastic thyroid cancer cell lines by activating c-Met and AKT.

## RESULTS

### Effect of the Shh pathway on thyroid tumor cell motility and invasiveness

**Figure 1:**
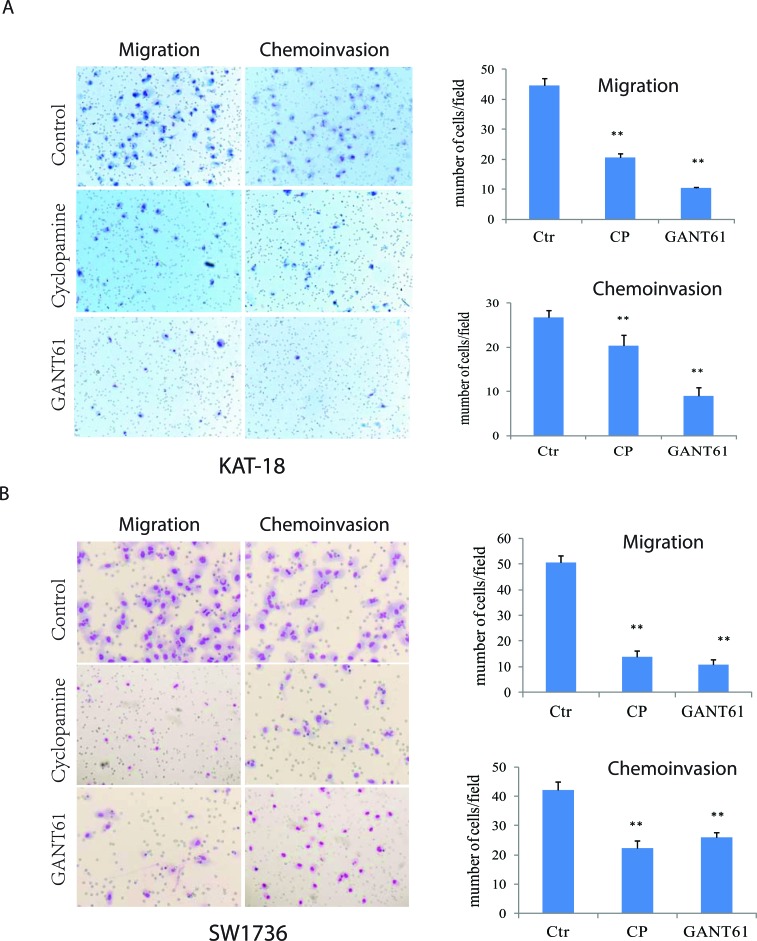
Inhibition of the Shh pathway leads to reduced cell motility and invasive potential KAT-18 **A.** and SW1736 **B.** seeded in uncoated or Matrigel-coated Boyden chambers in duplicate were incubated for 24 hr in the presence of 0.5% DMSO, cyclopamine (5 μM) or GANT61 (10 μM). Cells migrating through the pored PET membrane were stained in Diff-Quik solutions. Data represent the mean ± SD of the numbers of the cells in five random fields (10X) in duplicate. The experiment was repeated at least twice with similar results. *p < 0.05; **p < 0.01.

We first examined the effect of cyclopamine, a Smo inhibitor, and GANT61, a Gli1 inhibitor, on the motility and invasiveness of KAT-18 and SW1736 cells. As shown in Figure 1A, cyclopamine and GANT61 decreased KAT-18 cell motility by 54% and 76%, respectively, and inhibited the invasive potential of KAT-18 cells by 24% and 66%, respectively. Cyclopamine and GANT61 inhibited the motility of SW1736 cells by 73% and 79%, respectively, and inhibited the invasive potential of SW1736 cells by 47% and 38%, respectively (Figure 1B). According to our prior study [19], SW1736 cells are resistant to the anti-proliferative effect of cyclopamine, whereas KAT-18 cells are sensitive to cyclopamine, with the inhibition of cell proliferation by 50% after incubation 96 hr. The inhibitory effect of cyclopamine and GANT61 on cell motility and invasiveness was not due to their inhibitory effect on cell proliferation since the experiments were carried out overnight, the anti-proliferative effect after such a short duration is minimal and almost neglectable, in particular for SW1736 cells. KAT-18 and SW1736 cells were readily attached onto the uncoated or Matrigel-coated inserts in the absence or presence of cyclopamine or GANT61 2 hr after seeding. Neither cyclopamine nor GANT61 interfered with the adhesion of these two cell lines.

**Figure 2:**
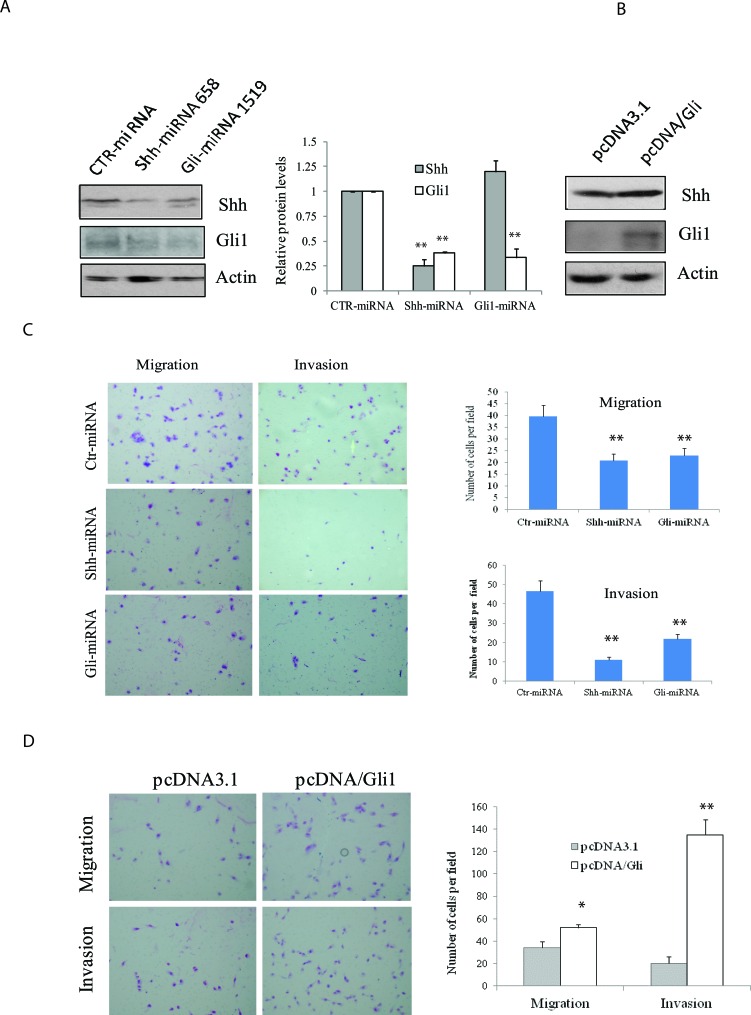
Effect of the Shh pathway knockdown or Gli1 overexpression on cell motility and invasiveness (A & B) KAT-18 cells stably transfected with an expression vector encoding a control, Shh or Gli1 miRNA **A.** or transfected with pcDNA3.1 or pcDNA/Gli1 **B.** were analyzed for Shh and Gli1 expression by Western blot with their specific antibodies. Actin was included as a loading control. The density of the bands was analyzed by using an NIH Image-J software and normalized by the arbitrary units of the density of actin. The results were the mean ± standard deviation from three independent experiments. C & D. Effect of the Shh pathway on cell motility and invasiveness. KAT-18 cells stably transfected with control miRNA, Shh-miRNA 658 or Gli-miRNA 1519 **C.** or KAT-18 cells transfected with pcDNA3.1 or pcDNA/Gli1 **D.** were seeded in uncoated or Matrigel-coated Boyden chambers and for their migratory and invasive potential after incubation for 24 hr. Data represent the mean ± SD of the numbers of the cells in five random fields (10X) in duplicate. Data represent the results of one of two experiments with similar results. *p < 0.05; **p < 0.01.

We reported earlier that two miRNA constructs targeting Shh and Gli1 were very effective at suppressing Shh and Gli1 expression in either transiently [19] or stably [23] transfected KAT-18 cells. We repeated Western blot analysis and again demonstrated the ability of these miRNA constructs to suppress Shh and Gli1 in KAT-18 cells stably transfected with these miRNA constructs (Figure 2A). Consistent with our earlier observation [23], we found that Shh knockdown in KAT-18 cells stably transfected with Shh-miRNA led to a modest decrease of Gli1 expression, probably due to an autocrine regulation by the Shh pathway (Figure 2A). Our recent study demonstrated the ability of pcDNA/Gli1 vector to overexpress Gli1 in stably transfected KAT-18 cells [23]. Again we confirmed Gli1 overexpression in pcDNA/Gli1-transfected KAT-18 cells (Figure 2B). Cell motility of Shh-miRNA- and Gli1-miRNA-transfected KAT-18 cells was decreased by 47% and 42%, respectively, and their invasive potential reduced by 76% and 53%, respectively (Figure 2C). In contrast, Gli1 overexpression in pcDNA/Gli1-transfected KAT-18 cells led to increased cell motility and invasiveness by 43% and 560%, respectively (Figure 2D).

### Induction of AKT and c-Met phosphorylation by the Shh pathway and the consequence on cell motility and invasiveness

**Figure 3:**
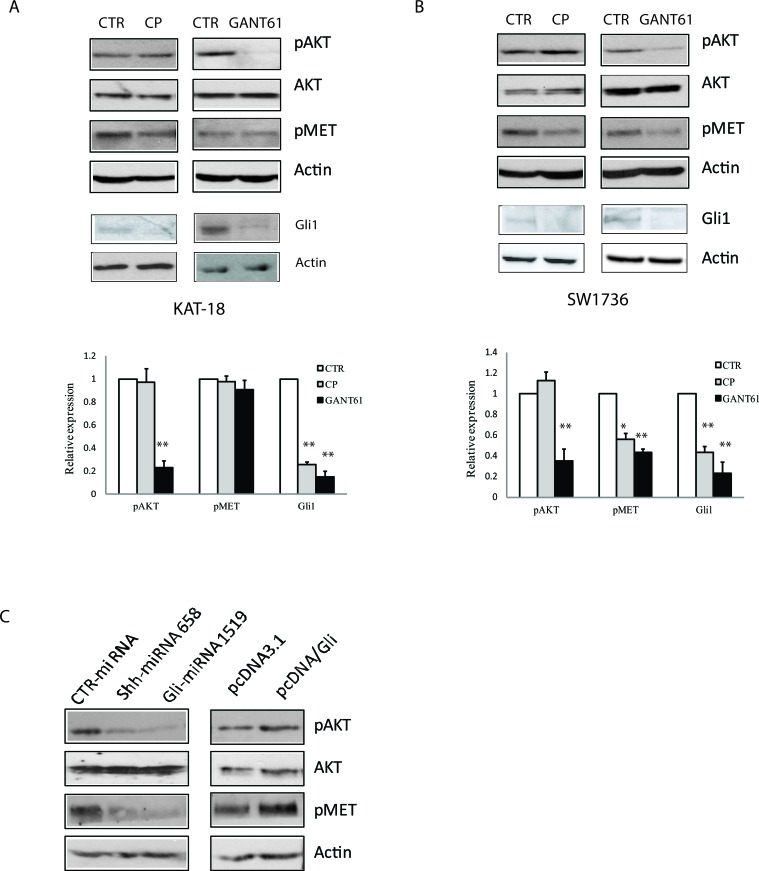
Effect of the Shh pathway on c-Met and AKT phosphorylation KAT-18 **A.** and SW1736 **B.** cells were treated with 0.5% DMSO, cyclopamine (5 μM) or GANT61 (10 μM) for 72 hr. The cells were harvested and analyzed for AKT (S473) phosphorylation, c-Met tyrosine phosphorylation at Y1230/1234/1235m and Gli1 expression by Western blot with their specific antibodies. The density of the bands from three independent experiments was analyzed and plotted in a bar graph. *p < 0.05; **p < 0.01. **C.** KAT-18 cells stably transfected with encoding a control, Shh or Gli1 miRNA or transfected with pcDNA3.1 or pcDNA/Gli1 were analyzed for AKT and c-Met phosphorylation by Western blot with their specific antibodies. Actin was included as a loading control.

The Shh pathway is implicated in promoting the cancer stem-like cell type of anaplastic thyroid cancer cell lines [23]. c-Met and AKT are highly phosphorylated and activated in invasive thyroid CSC [17]. Here we tested whether inhibition of the Shh pathway by two inhibitors led to decreased c-Met and AKT phosphorylation. Cyclopamine had little effect on c-Met and AKT phosphorylation in KAT-18 (Figure 3A) but did inhibit c-Met phosphorylation in SW1736 cells (Figure 3B). GANT61 significantly inhibited AKT phosphorylation at Ser-473 in both SW1736 and KAT-18 cells (Figure 3A and 3B). It also inhibited c-Met phosphorylation at tyrosine residues (Y1230/1234/1235) in SW1736 cells (Figure 3B) but had little effect in KAT-18 cells (Figure 3A). Cyclopamine and GANT61 both inhibited Gli1 expression in KAT-18 and SW1736 cells (Figure 3A and 3B). Shh and Gli1 knockdown significantly decreased AKT and c-Met phosphorylation (Figure 3C), whereas Gli1 overexpression modestly increased AKT and c-Met phosphorylation in KAT-18 cells, compared to pcDNA3.1-transfected cells (Figure 3C).

**Figure 4:**
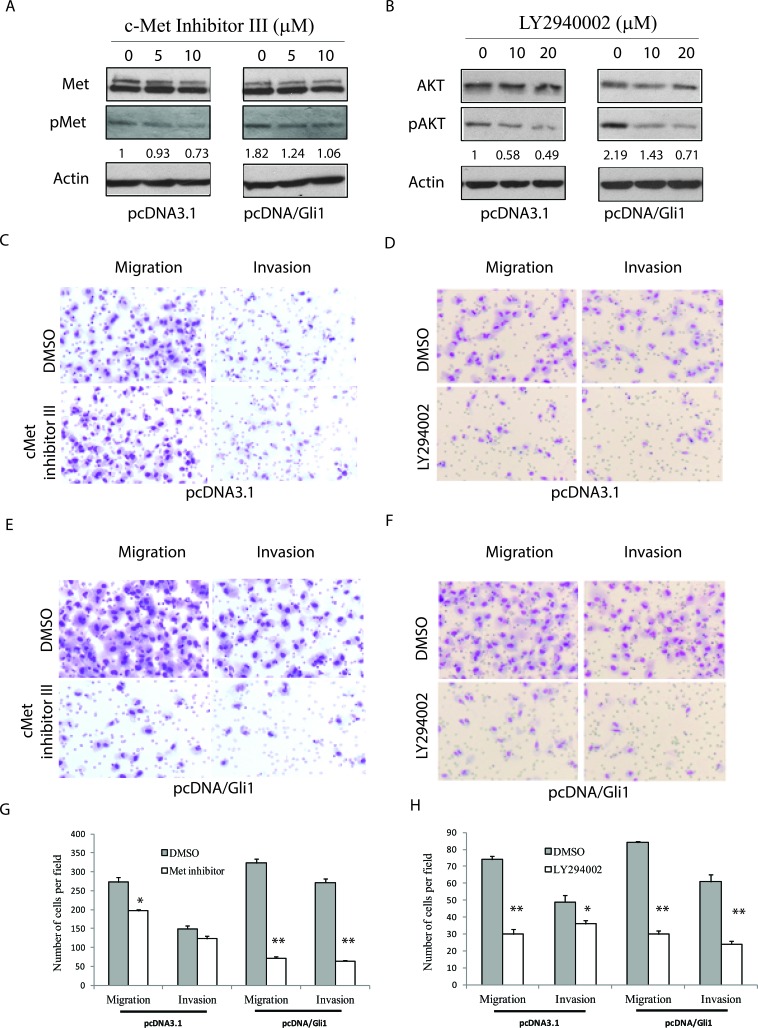
AKT and c-Met activation is required for the Shh pathway-induced cell motility and invasiveness pcDNA3.1 and pcDNA/Gli1-transfected KAT-18 cells seeded in 6-well plates were treated with a c-Met inhibitor III **A.** or an AKT inhibitor LY294002 **B.** with indicated concentrations for 24 hr. Cells were harvested and analyzed for c-Met tyrosine phosphorylation and AKT S473 phosphorylation by Western blot. The density of the bands was quantified by using an Image-J software and normalized by the arbitrary units of the density of actin. The numbers beneath the blots are a representative of two independent experiments with similar results. **C.**-**H.** Effect of c-Met inhibitor III and LY294002 on cell migration and invasiveness. pcDNA3.1 (C & D) and pcDNA/Gli1-transfected (E & F) KAT-18 cells seeded in Boyden chambers were incubated in the presence of DMSO (0.5%), c-Met inhibitor III (5 μM) (C & E) or LY294002 (10 μM) (D & F) for 24 hr. The cells migrating through the pored PET membrane were stained using Diff-Quik solution. The data represent the mean ± SD of the numbers of the cells in five random fields (10X) in duplicate (G & H). Experiments were repeated at least twice with similar results. The experiment was repeated twice with similar results. *p < 0.05; **p < 0.01.

We next determined if c-Met and AKT were required for the Shh pathway-induced cell motility and invasiveness. First, we found that the inhibitors of c-Met and AKT were able to inhibit c-Met tyrosine (Figure 4A) and AKT S473 (Figure 4B) phosphorylation in pcDNA3.1- and pcDNA/Gli1-transfected KAT-18 cells in a dose-dependent manner, respectively. c-Met kinase inhibitor III decreased the motility and invasiveness of pcDNA-transfected KAT-18 cells by 28% and 17% respectively, and inhibited the motility and invasive potential of Gli1-transfected KAT-18 cells by 78% and 77% respectively (Figure 4G). LY294002, an AKT inhibitor, decreased the motility and invasiveness of pcDNA-transfected KAT-18 cells by 60% and 27% respectively, and the motility and invasive potential of Gli1-transfected KAT-18 cells by 64% and 61%, respectively (Figure 4H). Of note, c-Met inhibitor III weakly inhibited KAT-18 cell migration but did not significantly inhibit invasiveness (Figure 4G). This is likely due to low basal level c-Met phosphorylation and activation in KAT-18 cells (Figure 6B)

### Role of Snail in thyroid tumor cell motility and invasiveness

**Figure 5:**
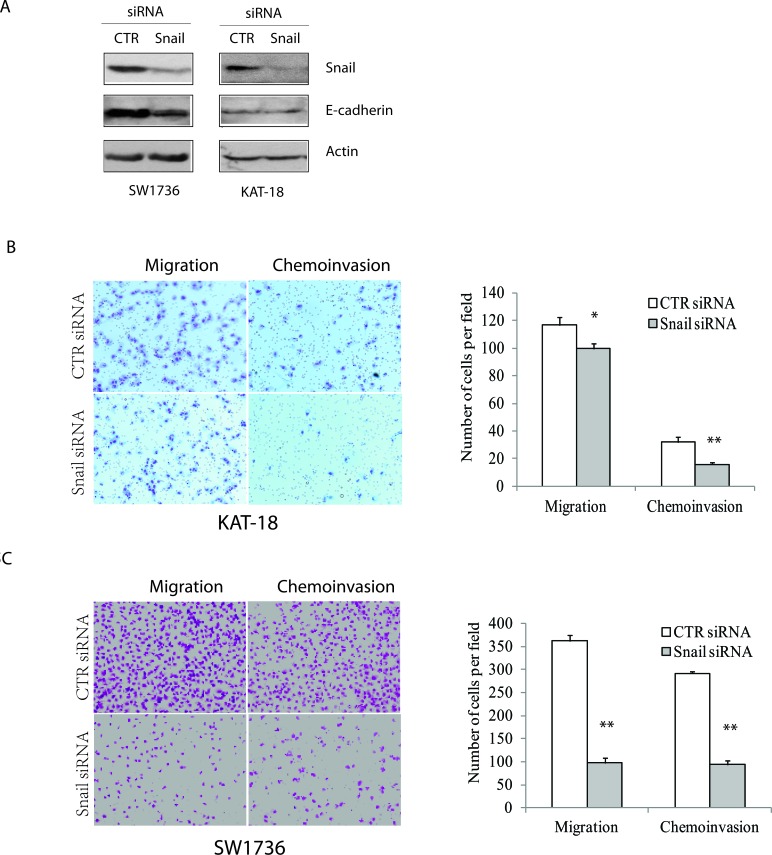
Snail knockdown inhibits cell motility and invasiveness KAT-18 and SW1736 cells seeded in 35-mm dishes were transfected with a scrambled control siRNA or Snail siRNA (2.5 nmole each). After incubation for 48 hr, the cells were harvested and analyzed for Snail and actin expression by Western blot **A.** or for cell motility and invasiveness of KAT-18 **B.** and SW1736 cells **C.** in uncoated or Matrigel-coated Boyden chamber. Data represent the results of one of two independent experiments with similar results. *p < 0.05; **p < 0.01.

**Figure 6:**
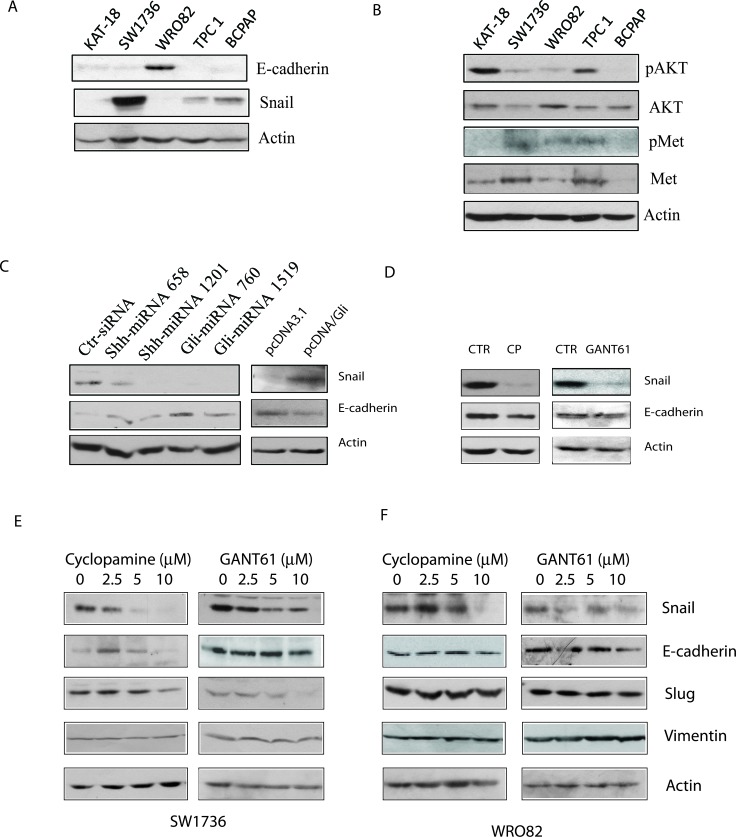
Effect of the Shh pathway on EMT **A.** & **B.** Cell lysates of KAT-18, SW1736, WRO82, TPC1, and BCPAP cell lines were analyzed for the expression of E-cadherin, Snail, and actin **A.** or pAKT, AKT, pMet, Met, and actin **B.** by Western blot. KAT-18 cells stably transfected with an expression vector encoding a control, Shh or Gli1 miRNA **C.** or transfected with pcDNA3.1 or pcDNA/Gli1 **C.** were analyzed for E-cadherin expression. (D-F) The effect of the Shh pathway inhibitors on E-cadherin expression. KAT-18 cells **D.** were treated with 0.5% DMSO as a vehicle control, cyclopamine (5 μM) or GANT61 (10 μM) for 72 hr. The cells were harvested and analyzed for E-cadherin and actin expression by Western blot with their specific antibodies. SW1736 **E.** and WRO82 **F.** cells were incubated in the presence of vehicle (0.5% DMSO) or the indicated concentrations of cyclopamine or GANT61 for 72 hr. The cells were harvested and analyzed for the expression of several genes involved in EMT.

Since Snail is tightly regulated by the Shh pathway, and Snail has been implicated in tumor cell metastasis in an EMT-dependent and –independent manner [24, 25], we tested if Snail was involved in regulating thyroid tumor cell motility and invasiveness. As shown in Figure 5A, Snail expression was effectively suppressed in KAT-18 and SW1736 cells transfected with Snail siRNA. Unexpectedly, Snail knockdown had no or little inhibitory effect on E-cadherin expression. Snail knockdown decreased KAT-18 cell motility and invasiveness by 15% and 50%, respectively (Figure 5B), and more effectively decreased SW1736 cell motility and invasiveness by 83% and 67%, respectively (Figure 5C). The differential sensitivity of KAT-18 and SW1736 cells to Snail suppression-mediated inhibition of cell motility and invasiveness is likely due to differential Snail expression in these two cell lines (Figure 6A).

### Role of the Shh pathway in EMT

We first tested if the Snail levels in 5 thyroid tumor cell lines inversely correlated with E-cadherin expression. Western blot analysis revealed that E-cadherin was expressed at high levels in WRO82 cells but at very low levels in four other cell lines (KAT-18, SW1736, TPC1, and BCPAP) (Figure 6A). Snail was expressed at relatively low levels in WRO82 cells but was expressed in four other cell lines, in particular in SW1736 cells. AKT was highly phosphorylated at S473 in KAT-18 cells, whereas c-Met tyrosine phosphorylation was very low in this cell line (Figure 6B). It appears that there was no correlation between Snail expression and AKT/c-Met phosphorylation (Figure 6A and 6B). Suppression of the Shh pathway by Shh and Gli1 knockdown weakly increased E-cadherin expression, whereas Gli1 overexpression slightly decreased E-cadherin expression (Figure 6C). Unexpectedly, cyclopamine and GANT61 slightly decreased or had no effect on E-cadherin expression (Figure 6D) in KAT-18 cells. Cyclopamine and GANT61 all drastically inhibited Snail expression in a dose-dependent manner in SW1736 cells (Figure 6E) but had relatively weak effect on WRO82 cells, probably due to very low level of Snail expression. These inhibitors had no or minimal effect on E-cadherin, Vimentin, and Slug expression in SW1736 and WRO82 cells (Figure 6E and 6F).

## DISCUSSION

Our present study showed that inhibition of the Shh pathway by Smo and Gli1 inhibitors or by Shh and Gli1 knockdown led to decreased cell motility and chemoinvasion in KAT-18 and SW1736 cells, whereas Gli1 overexpression enhanced KAT-18 motility and invasive potential. AKT and c-Met phosphorylation was increased in Gli1-transfected KAT-18 cells but decreased in KAT-18 cells with Shh and Gli1 knockdown. c-Met inhibitor III and the AKT inhibitor LY294002 decreased cell motility and invasiveness of Gli1-transfected cells. In particular, c-Met inhibitor III was more effective at inhibiting cell migration and invasion in Gli1-transfected than pcDNA3.1-transfected KAT-18 cells, probably as a result of increased c-Met activation in Gli1-transfected cells (Figure 4A). Our studies suggest that the Shh pathway promotes thyroid tumor cell motility and invasiveness by activating AKT and c-Met. Of note, our prior clinical study did not reveal a correlation between the Shh pathway activation and the invasive potential of thyroid cancer [19]. This is likely due to the non-canonical activation of Gli1 by other pathways such as the MAP kinase pathway due to RAS and BRAF gene mutations [26]. In addition, the activation of the PI-3 and MAP kinase pathways also induces the expression of thrombospondin-1 and EMT-related genes to promote tumor invasion [27-29]. Myopericytoma, a rare tumor with a phenotype of pluripotent stem-cell-like pericytes, is also highly aggressive with recurrent disease [30]. Targeting mutant B-Raf kinase in myopericytoma leads to the inhibition of cell migration, invasion, and angiogenesis [30].

**Figure 7:**
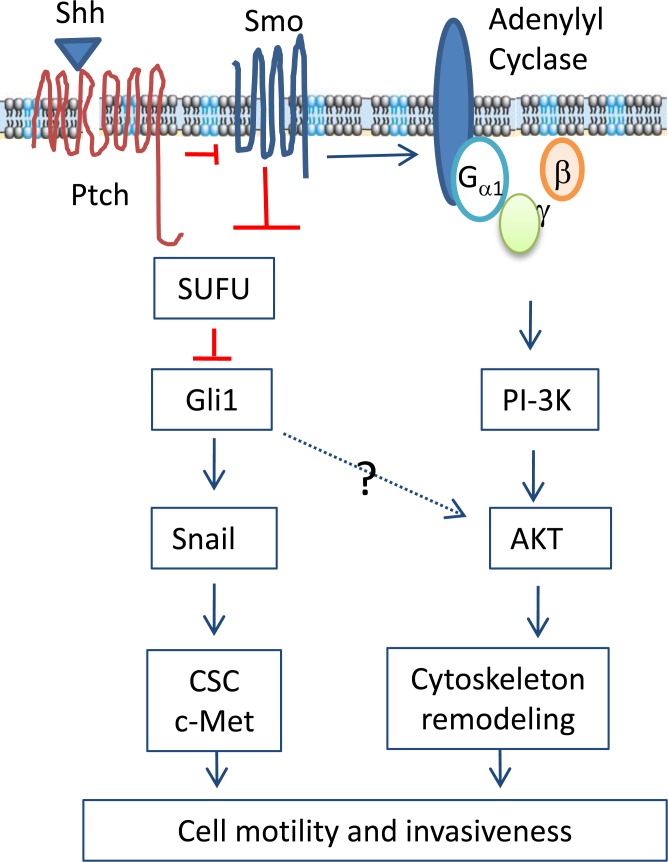
Mechanisms of the Shh pathway-induced cell motility and invasiveness The binding of Shh to the 12-pass transmembrane receptor Ptch leads to the translocation and activation of Smo, a G protein-coupled receptor. Smo activates Gli1 through inhibiting SUFU, an inhibitors of Gli1. In addition, Smo activation also leads to the formation of the G_β_ and G_γ_ heterodimer, which activates the PI-3-kinase pathway. AKT activation leads to cytoskeleton remodeling and cell motility. Snail expression can be transcriptionally induced by Gli1. The Shh pathway may also contribute to increased invasiveness by converting cells to cancer stem-like cell phenotype and c-Met activation.

Smo functions as a G protein-coupled receptor. Activation of Gβγ leads to the activation of PI-3 kinase, whereas inhibition of adenylyl cyclase leads to the inhibition of PKA and in part the activation of Gli1 (Figure 7) [11]. Indeed, N-Shh induces AKT phosphorylation in esophageal and gastric cancer cell lines [10, 31]. Cyclopamine inhibits AKT phosphorylation in hepacellular [13], esophageal [31], and gastric [10, 31] cancer cell lines. Similar to these observations, our study showed that Shh/Gli1 knockdown and GANT61 were very effective in suppressing AKT phosphorylation in KAT-18 and SW1736 cells, whereas Gli1 overexpression increased AKT phosphorylation in KAT-18 cells. Unexpectedly, we found that cyclopamine inhibited Gli1 expression but had little effect on AKT phosphorylation in KAT-18 and SW1736 cells (Figure 3A and 3B). How cyclopamine lacked the inhibitory effect on AKT phosphorylation is puzzling. One explanation is that many chemotherapeutic drugs are capable of activating the PI-3 and/or MAP kinase pathways [32]. It is not clear whether cyclopamine, a Smo inhibitor, may activate the PI-3 kinase pathway, thus counteracting its inhibitory effect on AKT phosphorylation mediated through inhibiting the Shh pathway. A second possibility is that Gli1 activation is responsible for AKT phosphorylation. Blocking the Smo activity by cyclopamine cannot prevent non-canonical Gli1 activation. Therefore a Smo inhbitor cannot, whereas a Gli1 inhibitor can inhibit AKT phosphorylation. In support of this notion, Srivastava et al. [15] recently reported that GANT61 inhibits AKT phosphorylation in embryonal and alveolar rhabdomyosarcomas.

We noticed that E-cadherin expression was not consistently regulated under different experimental settings. For example, overexpression of Gli in KAT-18 cells led to increased Snail expression and decreased E-cadherin expression. In contrast, Snail knockdown by siRNA did not increase E-cadherin expression in KAT-18 cells (Figure 5A). There are several explanations for this discrepancy: 1) basal level of Snail expression in KAT-18 cells was relatively low (Figure 6A). Indeed, Snail knockdown only weakly inhibited KAT-18 cell motility and invasiveness (Figure 5B); 2) Snail suppression by siRNA is transient and incomplete; 3) The E-cadherin promoter is probably highly methylated in anaplastic cell lines [33-35]. Transient suppression of Snail by siRNA is not sufficient to induce E-cadherin expression.

We also noticed that drug treatment did not recapitulate the effect of Shh and Gli1 knockdown on E-cadherin expression. There are several explanations again: 1) E-cadherin promoter is highly methylated [33-35]. Chronic stable expression of miRNAs that knock down Shh or Gli1 may alter the methylation status of the E-cadherin promoter. In contrast, drug treatments, similar to Snail siRNA, have only transient effect. 2) Nonspecificity of drugs: CP and GANT61, when used at a low concentration, actually increased E-cadherin expression. When they were used at high concentrations, their inhibitory effect on E-cadherin expression could be due to their off-target effect; 3) Regulation of E-cadherin expression is complex. Gli1 and Snail can positively and negatively regulate its promoter activity. Nevertheless, our data consistently showed that stable suppression of Shh and Gli1 expression with miRNA constructs increased E-cadherin expression, whereas Gli1 overexpression in pcDNA/Gli1 stably tranfected cells increased Snail expression and inhibited E-cadherin expression.

The mechanisms by which the Shh pathway stimulates cell motility and invasiveness are complex. Our present study demonstrated that AKT phosphorylation was increased by Gli1 overexpression and decreased by GANT61 or by Shh and Gli1 knockdown in KAT-18 cells. It is well established that Akt phosphorylates several cytoskeleton proteins such as filamin A, pappadin, and vimentin, and that the PI-3 kinase pathway stimulates cell motility in part by cytoskeleton remodeling [36]. Moreover, inhibition of PI-3 kinase by LY294002 led to the inhibition of thyroid tumor cell motility and invasion (Figure 4). We conclude that activation of the PI-3 kinase pathway plays a dominant role in the Shh pathway-induced cell motility and invasiveness. Consistent with our observations, inhibition of the PI-3 kinase pathway blocks the Shh-pathway-induced tumor cell invasiveness and metastasis of gastric cancer cells [37].

The Shh pathway may also promote tumor cell motility and invasiveness by inducing EMT, a cellular process best characterized by decreased expression of E-cadherin and increased expression of vimentin, fibronectin and N-cadherin. The Shh pathway promotes EMT by inducing Snail expression, leading to E-cadherin expression [38, 39]. For example, the Shh pathway induces EMT in ovarian and pancreatic cancers [40] [5]. Intriguingly, Gli1 itself is able to induce E-cadherin expression through the Gli1-binding site in the E-cadherin promoter [5, 6]. Thus, E-cadherin can be positively regulated by Gli1 but negatively regulated by Snail. The effect of the Shh pathway on E-cadherin expression is subject to the balance of Gli1 and Snail regulation. Our present study revealed that E-cadherin expression was weakly regulated in KAT-18 cells when the Shh pathway was persistently suppressed or activated through a genetic approach but minimally regulated in three thyroid tumor cell lines by transient inhibition with cyclopamine and GANT61. Liao et al. made similar observations in a SCOV3 ovarian cancer cell line [5]. In addition, we found that the Shh pathway had little effect on the expression of several other EMT-related genes such as vimentin, N-cadherin, and fibronectin. These results suggest that blockade of the Shh pathway was unable to reverse EMT in two anaplastic cell lines, and that the Shh pathway-induced cell motility and invasiveness is largely independent of EMT. Whether Shh pathway activation induces EMT in normal thyrocytes and in well differentiated thyroid tumor cell lines remains to be determined.

Todardo et al. [17] reported that c-Met is highly activated in thyroid cancer stem cells, and that inhibition of c-Met by gene knockdown significantly inhibits thyroid cancer stem cell motility and invasion. Our earlier study showed that SW1736 cells contain a much higher percent of thyroid cancer stem-like cells than that in KAT-18 cells [23]. In our present study, we found that cyclopamine had little effect on c-Met phosphorylation in KAT-18 cells but inhibited c-Met phosphorylation in SW1736 cells (Figure 3). Moreover, we found that c-Met inhibitor III had little effect on the migration and invasion of untransfected KAT-18 cells but inhibited the migration and invasions of Gli1-transfected KAT-18 cells (Figure 4). Lack of inhibitory effect by cyclopamine on c-Met phosphorylation and lack of inhibitory effect by the Met kinase inhibitor III on cell migration and invasion was likely due to lower c-Met expression (Figure 6) and lower percent of cancer stem-like cells in KAT-18 cells than that in SW1736 cells [23]. In contrast, we found that stable suppression of Shh expression in Shh-miRNA-transfected KAT-18 cells significantly decreased c-Met expression, whereas stable overexpression of Gli1 significantly increased c-Met expression. Differential effect of cyclopamine and Shh knockdown on c-Met and AKT activation as well as on E-cadherin expression could be due to the off-target effect of CP. Alternatively, stable suppression of the Shh pathway by gene knockdown may lead to c-Met and E-cadherin promoter demethylation.

In addition to its effect on EMT, Snail is capable of stimulating tumor cell motility and invasion by inducing the expression of matrix metalloproteinases (MMP). For example, Snail overexpression in pancreatic ductal adenocarcinomas increases tumor cell invasiveness by increasing the expression of MMP-14 and membrane type (MT)-1-MMP [41, 42]. Activation of the Shh pathway induces the expression of MMP-9 in pancreatic cancer cell lines [37] and MT1-MMP expression in ovarian cancer cell lines [5]. Our recent and present studies demonstrated the ability of the Shh pathway to regulate Snail expression, and Snail suppression inhibited KAT-18 and SW1736 cell motility and invasiveness. Thus, the Shh pathway may also stimulate thyroid tumor cell motility and invasiveness by Snail-induced proteinase expression (Figure 7).

In summary, our present study showed that the Shh pathway was involved in activating the PI-3 kinase pathway and promoting the motility and invasiveness of two anaplastic thyroid cancer cell lines. We further showed that Snail and the activation of the PI-3 kinase pathway as well as c-Met played a critical role in mediating the Shh pathway-induced thyroid tumor cell motility and invasiveness. The inhibitors of the Shh pathway may have potential to be developed as novel anti-thyroid cancer drugs to control anaplastic thyroid CSC-like phenotype, tumor invasiveness and metastasis.

## MATERIALS AND METHODS

### Cell lines and plasmid DNA

WRO82, KAT-18 and SW1736 cell lines were authenticated and reported earlier [19, 23]. Characteristics and stable transfection of KAT-18 cells with miRNAs targeting Shh and Gli1 or with Gli1 overexpression have been recently reported [23]. The passages of the three cell lines used in our studies are: WRO82 between 80-95 passages; KAT-18 between 28 and 40 passages; SW1736 between 10-25 passages.

### Cell motility and chemoinvasion assay

Single cell suspensions of KAT-18 and SW1736 cells were prepared and seeded in the top chamber of the 24-well Transwell inserts (2×10^4^/well) in serum-free medium supplemented with 0.1% bovine serum albumin. For the chemoinvasion assay, the inserts were pre-coated with Matrigel (100 μg/well; Roche Diagnostics Corp., Indianapolis, IN). The Transwell inserts were placed in a 24-well companion plate filled with 0.75 ml of complete RPMI1640 medium containing 10% fetal bovine serum. To analyze the effect of PI-3 kinase and c-Met inhibitors on cell motility and invasive potential, LY294002 (10 μM) (Cell Signaling Technology, Danvers, MA), or c-Met inhibitor III (5 μM) (EMD Millipore, Billerica, MA) was added into both top and bottom chambers. After incubation for 24 h, the cells in the inner side of top chamber were removed by wiping with cotton swabs. The effect of cyclopamine (LC Laboratories, Woburn, MA), a Smo inhibitor, and GANT61 (Sigma, St. Louis, MO), a Gli1 inhibitor, on cell motility and invasiveness were similarly conducted. Snail knockdown was carried out as described [23] and similarly analyzed for its effect on thyroid tumor cell motility and invasiveness, The cells that migrated through uncoated or Matrigel-coated polyethylene terephthalate (PET) membranes were stained with a Diff-Quik kit (Mercedes Medical, Sarasota, FL). The membrane was sliced out, mounted on slides and sealed with the mounting media. The cells in five random fields (10X) were counted under a light microscope.

### Western blot

Cell lysates were prepared as described [23] and analyzed for the expression of AKT, phospho-AKT^S473^, c-Met tyrosine phosphorylation at Y1230/1234/1235, Gli1, Snail, Slug, Vimentin, and E-cadherin (Cell Signaling Biotechnology, Inc., Danvers, MA). For loading control, β-actin was detected by a mouse monoclonal antibody (Santa Cruz Biotechnology Inc., San Diego, CA). The density of the bands was analyzed by using an Image-J software and normalized by actin. The results were the mean ± standard deviation from three independent experiments.

### Statistical analysis

The differences in the number of the cells migrating through the PET membrane between different treatment groups were statistically analyzed by using an unpaired Student *t* test. A *p* value of <0.05 was considered statistically significant. All statistics was performed with SigmaPlot 11 software (Systat Software, Inc, San Jose, CA).
